# Homoeologous gene silencing in tissue cultured wheat callus

**DOI:** 10.1186/1471-2156-9-65

**Published:** 2008-10-17

**Authors:** Andrew Bottley, Natalie H Chapman, Robert MD Koebner

**Affiliations:** 1 Department of Pharmacy, University Nottingham, NG7 2RD, UK; 2 Department of Plant Sciences, University of Nottingham, LE12 5RD, UK

## Abstract

**Background:**

In contrast to diploids, most polyploid plant species, which include the hexaploid bread wheat, possess an additional layer of epigenetic complexity. Several studies have demonstrated that polyploids are affected by homoeologous gene silencing, a process in which sub-genomic genomic copies are selectively transcriptionally inactivated. This form of silencing can be tissue specific and may be linked to developmental or stress responses.

**Results:**

Evidence was sought as to whether the frequency of homoeologous silencing in *in vitro *cultured wheat callus differ from that in differentiated organs, given that disorganized cells are associated with a globally lower level of DNA methylation. Using a reverse transcription PCR (RT-PCR) single strand conformation polymorphism (SSCP) platform to detect the pattern of expression of 20 homoeologous sets of single-copy genes known to be affected by this form of silencing in the root and/or leaf, we observed no silencing in any of the wheat callus tissue tested.

**Conclusion:**

Our results suggest that much of the homoeologous silencing observed in differentiated tissues is probably under epigenetic control, rather than being linked to genomic instability arising from allopolyploidization. This study reinforces the notion of plasticity in the wheat epi-genome.

## Background

In diploid species, the control of gene expression is largely under the control of promoters and transcription factors, whereas in polyploids such as hexaploid wheat or tetraploid cotton, an additional layer of complexity is created by epigenetic variation. Thus, there is a growing body of evidence showing that each homoeologous gene copy does not necessarily contribute equally to the global transcriptome [1,2]. In hexaploid wheat, for example, one of the three homoeologues normally present is not expressed in the leaf for approximately 30% of single copy genes, with a similar figure observed for roots [1]. This form of silencing has been shown to be variety-dependent [3]. In tetraploid cotton trichome cells, similarly around 30% of genes show a noticeable bias in expression level towards one genome [4], and, during development, this bias can be shifted. This form of transcriptional inactivation has been termed 'homoeologous gene silencing' and can be either tissue-specific or associated with a developmental process [5,6]. It is not known as yet how this silencing is either imposed or maintained, although it has been suggested to arise from genomic shock occurring at or shortly after a polyploidization event [7]. However, recent investigations based on synthetic *Arabidopsis *polyploids have shown that when methylation maintenance is compromised, some previously silenced genes become re-activated, thus implicating epigenetic control [8].

In a disorganized callus grown *in vitro*, chromosomal deletions, translocations, transposon movement and epigenetic repatterning all occur at a much higher level than *in planta *[9-12]. Among barley plants regenerated from tissue culture, both DNA sequence mutations and changed methylation patterns have been observed, with the latter occurring much more often than the former [13]. Similarly, in re-generated hop plants, "somaclonal" changes are more frequently epigenetic rather than genetic [14]. If, as has been suggested, methylation is responsible for much of the observed silencing in polyploids [8], and since callus cells are relatively demethylated compared to differentiated cells *in planta *[12,15,16], then the level of silencing present in callus cells would be predicted be lower than *in planta*. Thus, in this study, we set out to examine the pattern of homoeologous silencing in wheat callus cells. Our aim was to establish whether the undifferentiated state of these cells influenced the level of homoeologous silencing of genes known to be affected in this way in the root or the leaf.

## Results and Discussion

As the process of tissue culture is known to induce sequence re-arrangements and point mutations [17], we first compared the SSCP profiles of amplified callus genomic DNA (gDNA) to those of amplified cv. Florida gDNA. No detectable evidence for such events within the amplified sequences was noted in any of the material tested. Of the seven homoeologous sets of single-copy genes, in which at least one homoeologue is not expressed in cv. Florida leaf or root [3], four showed expression of all three homoeologues in the callus, while the other three were not expressed at all (Table 1). For instance, the A genome homoeologue of EST BE426364 is expressed in callus, but not expressed in the root tissue of cv. Florida and in ten of a sample of 16 other varieties [3]. In order to increase the number of genes sampled, we sampled a further nine genes, in which at least one homoeologue is not expressed in at least one tissue of cv. Chinese Spring [1]. Of these, all homoeologues were expressed in the callus, and the remaining one was not expressed. Among a further four genes showing full expression in cv. Chinese spring leaf and/or root were tested, all were also fully expressed in the callus (Table 1).

**Table 1:** A comparison between; genes shown to be expressed in leaf and/or root tissue of Chinese Spring (CS) (Bottley et al, 2006), the presence or absence of homoeologue silencing in the cultivar Florida (Bottley et al, 2008) and the expression of the same genes in callus tissue.

Genbank id	Expressed Callus	Homoeologue silenced in CS	Tissue	Homoeologues identifiable in CS	Homoeologue silenced in Florida	Putative function
BE399113	Y	D/D	LEAF/ROOT	B D	B	Unknown
BE444894	Y	D/B	LEAF/ROOT	A B D		saline responsive OSSRIII protein
BF482273	Y	D/B	LEAF/ROOT	B D		Unknown
BF201235	N	D	LEAF	A B D		Rubisco subunit binding-protein alpha subunit
BF473379	Y	D	LEAF	B D	D	Unknown
BF478825	Y	D	LEAF	A B D		Unknown
BF484100	N	D	LEAF	A B D	B	Unknown
BM138439	Y	D	ROOT	A B D		
BE443527	N	B/B	LEAF/ROOT	A B D	B	Unknown
BE404371	Y	B	ROOT	B D		NADH glutamate dehydrogenase
BE495400	N	B	ROOT	A B D	A	Unknown
BE499478	Y	B	ROOT	B +	B	FAT domain-containing protein/phosphatidylinositol 3- and 4-kinase family protein
BF202681	Y	B	ROOT	A B		Unknown
BE426364	Y	A/A	LEAF/ROOT	A D	A	glyceraldehyde-3-phosphate dehydrogenase
BE591763	Y	A	LEAF	A B D		Unknown
BF202265	Y	A	LEAF	A +		Unknown
BE500510	Y	-	-	-		
BE591372	Y	-	-	-		
BE638105	Y	-	-	-		
BM136908	Y	-	-	-		

The symbol '-' denotes that the homoeologous gene set is not afflicted by silencing. EST sequences blasted against NCBI Nucleotide collection

Homoeologous gene silencing, as understood to date, refers to events in genetically predictable and stable organs/tissues [reviewed by [18]]. This form of silencing has been successfully detected in cDNA populations generated from global RNA extracted from a range of complex multi-cell type organ such as a leaf, root, pistil, and spikes at the bolting stage [19,1,2,6]. In the disorganized callus, chromosomal deletions, translocations, transposon movement and epigenetic repatterning are enhanced [9-12]. DNA methylation levels in *in vitro *callus cells are proven to be highly variable [20] and in many instances lower than that in organized tissues, even though this difference, at a global level, is relatively small [12,18,16]. Demethylation has been demonstrated to relieve homoeologous silencing in polyploid Arabidopsis [8]. Therefore if methylation is responsible for this form of silencing in wheat, one plausible hypothesis is that the demethylation induced in the callus cells occurred at a relatively early stage of callus development, since otherwise the majority of cells would still be affected by silencing, and thus the detection of de-silencing would be compromised. In tobacco (*Nicotiana tabacum*) callus, rDNA genes become demethylated as early as 14 days after callus induction and a low level of methylation is stably maintained throughout prolonged culture [16].

## Conclusion

The pattern of homoeologous silencing in distinct tissues of wheat [6,1] or single cells of cotton [4] suggests the evolution of sub-functionalization of the expression of each homoeologue within a particular cell type or organ [4]. This form of silencing is typically highly specific, heritable and in some instances is associated with a particular developmental phase [2,1,3] suggesting a model in which silencing is strictly controlled. Therefore we propose that the absence of cellular control lifts the suppression of homoeologous expression, and we further speculate that the most likely mechanism of suppression is methylation.

## Methods

### Plant materials, RNA extraction and cDNA synthesis

The bread wheat cv. Florida was selected on the basis that it readily generates callus tissue when cultured *in vitro *(Perry, personal communication). Calli were generated in replicate from four individual scutella obtained from mature seed, as described by [21]. Calli were split after 21 days in culture, and harvested after 42 days growth. The tissue was snap frozen in liquid N_2_, and total RNA was extracted using Trizol™ reagent (Sigma), following the manufacturer's instructions. Crude RNA preparations were treated with DNAse (Amersham Bioscience) and phenol/chloroform extracted [22]. The presence/absence of contaminating genomic DNA was tested by amplification with a number of well-characterized PCR primers, and the quantity and quality of RNA were compared after agarose gel electrophoresis with a quantitative RNA standard (Ambion). cDNA was synthesised with Superscript II™ (Invitrogen), using oligo dT as the polyA primer and following the manufacturer's protocol. Newly synthesised cDNA was again tested by amplifying with intron-spanning PCR primers, since this allows a distinction between RNA-derived DNA and carry-through gDNA.

### EST selection, primer design, PCR amplification and SSCP analysis

Unigene ESTs mapping exclusively to a set of homoeologous genes located on one of wheat chromosome groups 1, 2, 3 or 7 were selected from among those examined by [1] on the basis that they generated clearly defined SSCP profiles and showed some silencing in either cv. Florida or cv. Chinese Spring [3,1]. Primer sequences were as described in [1]. The identity of the EST loci is listed in Table 1. cDNA was diluted 1:20, and 1 μl of this dilution was used as template for a 10 μl PCR, together with 5 μl Hotstar Master Mix™, 3.5 μl water and 0.25 μl of each primer (10 mM concentration). The amplification programme consisted of a 15 min/95°C Taq polymerase heat activation, which also served to denature the template, followed by 35 cycles of 95°C/30 s, 59°C/5 s and 72°C/60 s, and completed with a single extension step of 10 min at 72°C. Amplicons were electrophoretically separated by SSCP and visualised by silver staining, as described elsewhere [1].

### Pattern analysis

SSCP profiles generated from genomic DNA were compared with those derived from the equivalent cDNA template. Where all homoeologous copies identified in the gDNA template were also present in the cDNA profile, this was taken to imply full expression. Where, despite the known presence of a gDNA locus, no matching cDNA was detected, the relevant locus was scored as 'silenced'.

## Authors' contributions

AB carried out the molecular genetic studies and NC helped in the drafting of the manuscript. RK conceived of the study, and participated in its design and coordination. All authors have read and approved the final manuscript.
